# Association of Cardiovascular Mortality with Concurrent Coronary Artery Calcification and Physical Activity: A Cohort Study

**DOI:** 10.3390/medicina59030522

**Published:** 2023-03-07

**Authors:** Tae-Kyung Yoo, Sung-Ho Lee, Hye-Chang Rhim, Mi-Yeon Lee, Eun-Sun Cheong, Mi-Hae Seo, Ki-Chul Sung

**Affiliations:** 1 Department of Medicine, MetroWest Medical Center, Framingham, MA 01702, USA; 2 Division of Cardiology, Department of Internal Medicine, Kangbuk Samsung Hospital, Sungkyunkwan University School of Medicine, Seoul 03181, Republic of Korea; 3 Department of Physical Medicine and Rehabilitation, Harvard Medical School/Spaulding Rehabilitation Hospital, Boston, MA 02129, USA; 4 Division of Biostatistics, Department of R&D Management, Kangbuk Samsung Hospital, Sungkyunkwan University School of Medicine, Seoul 03181, Republic of Korea; 5 Division of Cardiology, Department of Internal Medicine, Seoul Eulji Hospital, Eulji University School of Medicine, Seoul 06047, Republic of Korea; 6 Department of Internal Medicine, Soonchunhyang University Gumi Hospital, Gumi 39371, Republic of Korea

**Keywords:** coronary artery calcification, cardiovascular mortality, physical activity

## Abstract

*Background*: Increased coronary artery calcification (CAC) has been reported in individuals with high levels of physical activity (PA). However, the association between increased CAC in a physically active population and cardiovascular mortality has not yet been well-established. This study aimed to investigate the association between PA levels and the presence or absence of CAC and cardiovascular mortality. *Methods*: A cohort study was conducted from 1 January 2011 to 30 December 2019. Mortality data were updated until 30 December 2020. The study population comprised 56,469 individuals who had completed the International Physical Activity Short Form Questionnaire and had undergone CAC score evaluation using a CT scan. We divided the participants into four groups: physically inactive individuals without CAC, physically inactive individuals with CAC, moderately active and health-enhancing physically active (HEPA) individuals without CAC, and moderately active and HEPA individuals with CAC. The primary outcome was cardiovascular mortality. The Cox proportional hazard model with confounding factor adjustment was conducted. Inverse probability of treatment weighting-based marginal-structural modelling was conducted. *Results*: The median follow-up duration was 6.60 years. The mean (SD) age of the study participants was 41.67 (±10.91) years, with 76.78% (*n* = 43,359) men. Compared with individuals without CAC, individuals with CAC demonstrated higher cardiovascular disease mortality regardless of PA level (Inactive and CAC > 0, HR 2.81, 95% CI: 1.76–19.19; moderately active and HEPA HR 3.27, 95% CI: 1.14–9.38). *Conclusions*: The presence of CAC might be associated with cardiovascular mortality regardless of PA level.

## 1. Introduction

It is well known that physical activity improves and prevents metabolic abnormalities; it also prevents cardiovascular disease (CVD) and decreases CVD mortality [1,2,3]. Moreover, many studies have shown that physical activity increases overall life expectancy and CVD-free life expectancy [4,5]. Owing to its well-known benefits, the American Heart Association and the World Health Organization recommend at least 150 min per week of accumulated moderate-intensity or 75 min per week of vigorous-intensity aerobic physical activity [6,7]. The mechanism of action underlying the benefit of exercise is thought to involve decreasing sympathetic activation, arterial pressure, and heart rate; decreasing reactive oxygen species and inflammatory cytokines; and enhancing blood flow and endothelial NO production, which causes dilation of vessels [8]. However, many aspects of this mechanism are unknown and require further elucidation [9].

Coronary artery calcification (CAC) is a prevalent condition in older adults, occurring in >90% of men and 67% of women over 70 years of age [10]. CAC is important because it is a known marker of atherosclerosis [11]. CAC was once considered a quiescent disease process; however, it is now regarded as an active inflammatory process associated with atherosclerosis [12]. The presence of CAC is related to coronary artery disease regardless of risk factors or symptoms [13,14]. As this process occurs even when a patient remains asymptomatic, early diagnosis of CAC is considered important [10]. Measurement of a coronary artery calcium score with computed tomography has been used to measure CAC [15]. This has been proven to predict coronary heart disease events in an asymptomatic population [15].

To date, only a few studies have evaluated whether CAC among highly physically active populations eventually leads to cardiovascular mortality, and previous studies were mostly conducted on elite athletes [16,17]. Those results from “athlete’s heart” is difficult to apply to the general population, as the clinical profile of athlete’s heart is different from the general population due to circulatory and cardiac morphological alterations [18]. Therefore, we aimed to assess the association between physical activity, CAC, and CVD mortality in a general population. We hypothesized that the presence of coronary artery calcification might be associated with CVD mortality in the general population, regardless of physical activity level.

## 2. Methods

### 2.1. Study Population

Data from the Kangbuk Samsung Health Study (KSHS) was used for our analysis. The KSHS is a cohort study of the Korean population aged ≥18 years who underwent annual or biennial comprehensive health examinations at Kangbuk Samsung Hospital Total Healthcare Centers in Seoul and Suwon, Republic of Korea. Most participants were employees or the spouses of employees in the public sector or private companies. The study population comprised 56,469 individuals who had completed the International Physical Activity Short Form questionnaire (IPAQ-SF) and CAC score evaluation for analysis between 1 January 2011 and 30 December 2019. Participants’ mortality data were updated on 31 December 2020. This study was approved by the Institutional Review Board of Kangbuk Samsung Hospital (IRB No.: 2015-12-004-017). The requirement for informed consent was waived because only non-identifiable data obtained during the health screening examinations were used.

### 2.2. Clinical Status

At each health check-up, the study participants completed a detailed, standardized questionnaire to assess their lifestyles and related history, including medical history, medication use, family history, smoking habits, alcohol intake, physical activity, and education level. Smoking was categorized as never, former, and current smoker [17,19]. High alcohol intake was defined as alcohol intake >20 g/day for women and >30 g/day for men [20]. Education level was categorized as high school graduate or lower, college, or university graduate. A college, university graduate or higher level of education was defined as higher education. A family history of CVD was defined as a self-reported stroke or heart disease in a first-degree relative. A history of CVD was defined when participants answered “yes” to one of the questions, “Have you ever been medically diagnosed with stroke?”, “Have you ever been medically diagnosed with angina/myocardial infarction?”. The presence of peripheral arterial disease was not specifically asked. Body mass index (BMI), height, and weight were measured at each health checkup by trained medical staff. BMI was calculated by the formula; weight was divided by height squared (kg/m^2^). Hypertension was defined at the screening visit as a self-reported history of hypertension or self-reported use of antihypertensive medications, or a systolic blood pressure ≥140 mmHg or diastolic blood pressure ≥90 mmHg. Diabetes was defined as a self-reported history of diabetes or self-reported use of oral hypoglycemic agents or insulin or measured fasting serum glucose level ≥126 mg/dL or HbA1c level ≥6.5%.

During the health examination, trained medical personnel collected anthropometric data, including weight, height, and blood pressure, in a standardized manner. Participants were asked to rest for at least 10 min before the blood pressure measurement. Blood pressure was measured on the arm positioned at heart level using an automated oscillometric device (53000, Welch Allyn, Skaneateles Falls, NY, USA). Blood samples were collected after at least 8 h of fasting. All laboratory analyses were performed by the Laboratory Medicine Department at Kangbuk Samsung Hospital, which is accredited by the Korean Association of Quality Assurance for Clinical Laboratories and the Korean Society of Laboratory Medicine.

### 2.3. Assessment of Physical Activity

We assessed the participants’ physical activity levels using the validated Korean version of the IPAQ-SF [21]. The IPAQ-SF measures the weekly frequency of physical activities (moderate-to-high intensity) performed across any context, including work, home, and leisure, for over 10 consecutive minutes. Participants were divided into three groups according to the IPAQ-SF: inactive, moderately active (≥3 days of high-intensity activity of at least 20 min/day, ≥5 days of moderate-intensity activity for at least 30 min/day or ≥5 days of any combination of moderate- or high-intensity activities achieving at least 600 metabolic equivalents (MET)-min/week), and health-enhancing physically active (HEPA; ≥3 days of high-intensity activity accumulating more than 1500 MET-min/week or 7 days of any combination of walking, moderate- or high-intensity activities achieving at least 3000 MET-min/week) [21,22,23,24]. 

### 2.4. Coronary Artery Calcium Score and Cardiovascular Mortality Data

We used a Lightspeed VCT XTe-64 slice MDCT scanner (GE Healthcare, Tokyo, Japan) with a standardized protocol of 40 × 2.5 mm slice collimation, 400 ms rotation time, 120 kV tube voltage, and 124 mAS (310 mA × 0.4 s) tube current under electrocardiographically (ECG)-gated dose modulation to measure CAC. The Agatston method was used to calculate CAC [25,26]. The intraclass correlation coefficient for the CAC was 0.99. Participants were categorized into those with (CAC > 0) and without CAC (CAC = 0) groups, as a previous study reported that CVD mortality significantly increased from CAC > 0 [27]. 

Cardiovascular mortality was assessed using Korean National Statistical Office (KNSO) data. KNSO is a civil registry collecting all the data about the age, causes, date, and time of death. Physicians in Korea are mandated to report the death to the local government by law. KNOS retrieves this information from the local government and establishes a validated registry. The cause of death is reviewed, classified, and coded according to the World Health Organization definition and ICD-10. Almost 100% of deaths were certified by 2007 [28,29]. 

### 2.5. Statistical Analysis

As our study aimed to assess the relationship between baseline physical activity level, CAC, and CVD mortality, we analyzed data from an initial health examination. The CAC values were transformed into a categorical variable for the analysis (CAC = 0 or CAC > 0). Continuous variables with normal distribution were expressed as mean and standard deviation. Logarithmic transformation was performed for the creatinine variable due to its non-normal distribution. One-way analysis of variance (ANOVA) and independent sample *t*-tests were used to compare continuous variables between groups. Pearson’s chi-squared test was used to compare categorical variables, which are expressed as percentages. We further conducted a multivariable Cox proportional hazard analysis to estimate the hazard ratios (HRs) with a 95% confidence interval (CI) for mortality rate (per 10^5^ person-year (PY)), according to the physical activity level and CAC scores. We performed analyses adjusting for confounding factors, such as age and sex (Model 1). We further adjusted for confounding factors, such as sex, age, BMI, alcohol intake, smoking status, education level, systolic blood pressure, fasting glucose, serum low-density lipoprotein (LDL), and a history of heart disease, diabetes, dyslipidemia, and hypertension (Model 2). Lastly, we conducted marginal structural modelling to decrease bias from covariates [30]. Inverse probability of treatment weighting (IPTW) was estimated using Model 2. Stata version 17.0 (StataCorp LP, College Station, TX, USA) was used for all statistical analyses. The significance level was set at *p* < 0.05.

## 3. Results

### 3.1. Baseline Characteristics

The mean follow-up duration was 6.14 ± 2.72 years, with a median follow-up of 6.60 years. A total of 34 deaths were reported during follow-up. The mean (SD) age of the study participants was 41.67 (±10.91) years. The proportions of inactive, moderately active, and HEPA participants were 45.36% (*n* = 25,612), 36.47% (*n* = 20,597), and 18.17% (*n* = 10,260), respectively.

Those in the HEPA group were significantly older and had higher BMI, systolic blood pressure, fasting glucose, high alcohol consumption, lower smoking rate, higher rate of diabetes, dyslipidemia, hypertension, and history of CVD (fasting glucose, *p* = 0.004; history of CVD, *p* = 0.003; all other variables mentioned above, *p* < 0.001). Our study cohort showed a significantly higher proportion of participants with CAC > 0 in the HEPA group than in the inactive and moderately active populations (*p* < 0.001, *p*_trend_ < 0.001) (Table 1).

### 3.2. Mortality Based on CAC

Ten deaths occurred in the CAC = 0 group, whereas 24 CVD mortalities occurred in the CAC > 0 group. After adjusting for sex and age, the CAC > 0 group showed significantly higher CVD mortality rate than the CAC = 0 group (Model 1, HR 6.25, 95% CI 2.66–14.71). This trend remained true after further adjustment for BMI, alcohol intake, smoking status, education level, history of CVD, diabetes, dyslipidemia, hypertension, systolic blood pressure, fasting blood glucose, and serum LDL level (Model 2, HR 5.59, 95% CI 2.27–13.79). In the IPTW model, the CAC > 0 group was associated with increased CVD mortality (HR 7.13, 95% CI 2.87–17.71) (Table 2).

### 3.3. Mortality Rates—Based on Physical Activity Level and CAC

We assessed mortality rate based on physical activity and CAC levels. First, we categorized participants based on their physical activity. In the physically inactive group, those in the CAC > 0 group showed a higher CVD mortality rate than those in the CAC = 0 group after adjusting for age and sex (Model 1, HR 3.52, 95% CI 1.16–10.65). This relationship remained the same after further adjustment (Model 2, HR 3.68, 95% CI 1.14–11.90; IPTW, HR 5.81, 95% CI 1.76–19.21). In the moderately active and HEPA groups, those in the CAC > 0 group showed higher CVD mortality rate than those in the CAC = 0 group after adjusting for age and sex (Model 1, HR 16.82, 95% CI 3.39–83.58) and after further adjustment (Model 2, HR 12.63, 95% CI 2.37–67.10; IPTW HR 11.72, 95% CI 2.23–61.65) (Table 3).

We then categorized participants based on their CAC level. In the CAC = 0 group, those in the moderately active and HEPA groups showed significantly decreased mortality than those in the physically inactive group, with age and sex adjustment (Model 1, HR 0.02, 95% CI 0.04–0.95). This remained true after further adjustment, but there was no significance in IPTW model (Model 2, 0.19, 95% CI 0.04–0.90; IPTW, HR 0.28, 95% CI 0.05–1.47). In the CAC > 0 group, the moderately active and HEPA groups did not show any mortality difference in the age- and sex-adjusted model (Model 1, HR 0.81, 95% CI 0.36–1.84), and after full adjustment (Model 2, HR 0.77, 95% CI 0.33–1.76; IPTW, HR 0.56, 95% CI 0.17–1.89).

### 3.4. Comparison of Mortality Rates in Physically Inactive Participants without CAC as a Reference

Finally, we categorized the participants into four groups: physically inactive without CAC (CAC = 0), physically inactive with CAC (CAC > 0), moderately active and HEPA without CAC (CAC = 0), moderately active and HEPA with CAC (CAC > 0). Compared to physically inactive individuals without CAC, physically inactive individuals with CAC showed higher mortality rates (Model 2, HR 3.60, 95% CI 1.25–10.37; IPTW, HR 2.81, 95% CI 1.76–19.19). Moderately active and HEPA individuals without CAC demonstrated lower CVD mortality rates (Model 2: HR 0.20, 95% CI: 0.04–0.94). This significance disappeared in IPTW model, but there was a decreasing trend in CVD mortality in moderately active and HEPA without CAC group (IPTW, HR 0.28, 95% CI 0.05–1.45). Lastly, the moderately active and HEPA with CAC group was associated with increased CVD mortality in the IPTW model (IPTW: HR 3.27, 95% CI: 1.14–9.38) (Table 4).

## 4. Discussion

To the best of our knowledge, this is the first study to assess the association between physical activity, CAC, and CVD mortality in a relatively young Asian population. Our results showed that the physically active population had higher CAC scores. In the same physical activity level, the CAC > 0 group showed higher CVD mortality. In the same CAC level, there was a trend in decreased CVD mortality in physically active participants. Compared to the physically inactive without CAC group, both physically inactive with CAC and the moderately active and HEPA with CAC groups showed an increase in CVD mortality. Our results suggest that the presence of CAC might be associated with increased CVD mortality regardless of physical activity level. In addition, CAC may be a stronger driving factor on CVD mortality than physical activity level.

Recent studies have shown an association between long-term high-intensity physical activity and coronary artery calcification and atherosclerotic plaques [16,23,31,32,33]. This finding led to a new term, “coronary artery paradox” [34].

A prior study evaluating CAC among 152 endurance athletes showed a higher incidence of CAC > 300 (Agatston unit [AU]) and a larger number of atherosclerotic coronary plaques among male athletes than among physically inactive men [32]. Interestingly, the plaques of athletes showed a calcified and stable nature [32]. Another study that assessed the relationship between lifelong exercise volume and CAC among 284 athletes showed similar results [31,32]. These findings suggest that the CAC score per se, without considering the components of coronary plaques, may not be a useful marker of CVD in the physically active population. However, both studies assessed only athletes, one of the studies evaluated the male population only, and the studies did not assess CVD mortality directly [31,32].

Few prior studies have evaluated the association between CVD mortality, physical activity, and CAC. [16,35] One study of 21,758 men showed an increased CAC prevalence in the most physically active group. Meanwhile, CVD mortality in the most physically active with high CAC group (CAC ≥100 AU) did not show a significant CVD mortality difference between the physically less active and low CAC group (CAC < 100 AU) [16]. This study assessed only a male, predominantly white population, which limits its generalizability.

Another cross-sectional study of 3393 multiethnic participants assessed the relationship between recreational physical activity and CVD events, including CVD mortality [35]. In this study, recreational physical activity showed a significant inverse relationship with CVD, and the relationship was independent of CAC [35]. 

Currently, the Agatston score is the standard method to calculate the CAC score, which is weighted upwards for greater CAC density [25]. However, a previous study demonstrated an inverse association between CVD risk and the CAC density, and CAC volume was more predictive of CVD events [36]. To date, multiple studies have consistently reported that physical activity can increase CAC by increasing plaque calcification, but not CAC volume. These studies highlight the need for a new and precise approach that can differentiate between vulnerable and “stable plaques” for better predictability within the same CAC score in a physically active population [31,32,35]. 

Though multiple studies have suggested increased CAC in a physically active population, the cross-effect of physical activity and CAC on CVD mortality is unclear [23]. Our result suggests that regardless of physical activity level, the presence of CAC is associated with increased CVD mortality. In addition, it suggests CAC might be a stronger driving factor on CVD mortality than physical activity level.

Our study cohort is unique because we enrolled large numbers of men and women (n = 56,456; male, 76.79%; female, 23.21%), which strengthens the generalizability of the findings. Moreover, our study population incorporated the general population and was not limited to athletes. Lastly, we performed adjustments for known confounding factors for CVD, including education level and traditional CVD risk factors [6,37,38,39]. Previous studies did not consider education level as a confounding factor for CVD mortality [16,35], although there is evidence to suggest that education level, one of the markers of socioeconomic status, has an inverse relationship with CVD risk [39,40]. 

Despite these strengths, our study has several limitations. First, our study was performed in a relatively young, healthy Korean population, limiting the application of our results to other age groups and ethnicities. However, the young age of the participants could also be seen as a strength, since our study population was less affected by age or other comorbidities that can affect CVD mortality. Second, owing to the young population, the number of death outcomes was small. This small amount of mortality is the most important limitation of our study, which could have affected statistical significance. In addition, we could not further stratify the outcome according to the CAC level due to the small number of outcomes. However, previous studies have reported that the presence of CAC is associated with increased CVD mortality, while CAC = 0 can identify a group of asymptomatic subjects at very low cardiovascular risk [41]. Therefore, by stratifying the participants into two groups (CAC = 0 and CAC > 0), we could analyze the participants in low cardiovascular risk group and the elevated cardiovascular risk group [41]. Furthermore, we tried to overcome the small number outcome by performing marginal structural modelling. Future studies of more diverse ethnicities and age groups should be conducted to validate our results. Third, our data did not report the number of non-fatal myocardial infarctions, which is also important to consider in terms of the safety of a physically active population. Fourth, we used self-reports of physical activity. However, IPAQ-SF is a validated form to assess physical activity, and it is known to be well suited to conduct large population studies to assess physical activity among adults [21,42]. Fifth, as the study aim was to assess CVD mortality according to physical activity and CAC, our study did not report other causes of mortality and all-cause mortality. Sixth, since CAC and CVD mortality are age-dependent, the age covariate could introduce a bias. This could have affected the study result. Seventh, some of the lifestyle variables could have changed over time. Lastly, even though the size of difference was small, the HEPA group in our cohorts had a higher age, BMI, and a higher rate of diabetes. However, we adjusted those factors during the statistical analysis to minimize the effect of different characteristics in each group. Due to these limitations, our study result needs a cautious interpretation.

## 5. Conclusions

In conclusion, the presence of CAC might be associated with increased CVD mortality regardless of physical activity level. In addition, CAC might be a stronger driving factor for CVD mortality than physical activity level. Further prospective studies are needed to elucidate the exact relationship between exercise level and CAC, and CVD mortality.

## Figures and Tables

**Table 1:** Baseline characteristics of participants according to physical activity levels.

	Inactive	Moderate	HEPA	*p* Value	*p* for Trend
Number	25,612 (45.36)	20,597 (36.47)	10,260 (18.17)		
Age, years	41.18 ± 10.4	41.19 ± 10.7	43.85 ± 12.24	<0.001	<0.001
Men, %	18,851 (73.6)	16,562 (80.41)	7946 (77.45)	<0.001	<0.001
Current smoker, %	6595 (25.75)	4833 (23.46)	2145 (20.91)	<0.001	<0.001
High alcohol intake, %	4966 (19.39)	3764 (18.27)	2192 (21.36)	<0.001	<0.001
Higher education, %	18,146 (70.85)	15,401 (74.77)	6562 (63.96)	<0.001	<0.001
BMI, kg/m^2^	24.43 ± 3.5	24.58 ± 3.32	24.62 ± 3.17	<0.001	<0.001
SBP, mmHg	113.58 ± 12.85	114.49 ± 12.69	115.61 ± 12.74	<0.001	<0.001
Fasting glucose, mg/dL	98.43 ± 18.19	98.55 ± 17.69	99.11 ± 17.19	0.004	0.002
LDL, mg/dL	129.85 ± 34.22	129.55 ± 33.33	127.03 ± 33.83	<0.001	<0.001
Creatinine	0.89 ± 0.22	0.92 ± 0.17	0.91 ± 0.18	<0.001	<0.001
eGFR, mdrd	96.54 ± 17.21	95.03 ± 16.18	93.85 ± 16.56	<0.001	<0.001
Diabetes, %	1762 (6.88)	1464 (7.11)	874 (8.52)	<0.001	<0.001
History of CVD, %	291 (1.14)	240 (1.17)	160 (1.56)	0.003	<0.001
Dyslipidemia%	5353 (20.9)	4233 (20.55)	2168 (21.13)	0.449	<0.001
History of HTN, %	3751 (14.65)	3218 (15.62)	1980 (19.3)	<0.001	<0.001
CAC (AU)				<0.001	<0.001
0	21,949 (85.7)	17,268 (83.84)	8025 (78.22)		
>0	3663 (14.3)	3329 (16.16)	2235 (21.78)		

Values are expressed as the mean ±standard deviation, or percentage. Abbreviations: AU, Agatston unit; CAC, coronary artery calcification; BMI, body mass index; LDL, low density lipoprotein; eGFR, estimated glomerular filtration rate; MDRD, Modification of Diet in Renal Disease Study; SBP, systolic blood pressure; CVD, cardiovascular disease; HEPA, health-enhancing physical activity; HTN, hypertension.

**Table 2:** Mortality rate based on CAC.

	Number	Person-Years	Mortality (n)	Morality Rate (per 10^5^ PY)	Unweighted HR (95% CI)		IPTW HR (95% CI)
					Model 1	Model 2	
CAC (AU, 1 unit increase)	56,469	354,585.17	34	9.59 (6.85–13.42)	1.0008 (1.0003–1.0013)	1.0006 (1.0001–1.0012)	1.0007 (1.0003–1.001)
CAC = 0	47,242 (83.66)	30,2516.41	10	3.31 (1.78–6.14)	1 (reference)	1 (reference)	1 (reference)
CAC > 0	9227 (16.34)	52,068.76	24	46.09 (30.89–68.77)	6.25 (2.66–14.71)	5.59 (2.27–13.79)	7.13 (2.87–17.71)

Abbreviations: AU, Agatston unit; CAC, Coronary artery calcium; CI: confidence interval; HR: hazard ratio; IPTW: inverse probability of treatment weighting; PY, person-year.Model 1: adjusted for age, sex; Model 2: adjusted for sex, age, BMI, alcohol intake, smoking status, education level, systolic blood pressure, fasting glucose, serum LDL, history of heart disease, diabetes, dyslipidemia, and hypertension. IPTW: marginal structural model based on IPTW.

**Table 3:** Mortality rates based on physical activity levels and CAC.

	Number	Person-Years	Mortality (*n*)	Morality Rate (per 10^5^ PY)	Model 1	Model 2	IPTW HR (95% CI)
Inactive	25,612	159,992.06	18	11.25 (7.09–17.86)			
CAC = 0	21,949 (85.70)	139,454.32	8	5.74 (2.87–11.47)	1 (reference)	1 (reference)	1 (reference)
CAC > 0	3663 (14.30)	20,537.73	10	48.69 (26.2–90.49)	3.52 (1.16–10.65)	3.68 (1.14–11.90)	5.81 (1.76–19.21)
Mod active + HEPA	30,857	194,593.11	16	8.22 (5.04–13.42)			
CAC = 0	25,293 (81.97)	163,062.08	2	1.23 (0.31–4.9)	1 (reference)	1 (reference)	1 (reference)
CAC > 0	5564 (18.03)	31,531.03	14	44.4 (26.3–74.97)	16.82 (3.39–83.58)	12.63 (2.37–67.1)	11.72 (2.23–61.65)
CAC = 0	47,242	302,516.41	10	3.31 (1.78–6.14)			
Inactive	21,949 (46.46)	139,454.32	8	5.74 (2.87–11.47)	1 (reference)	1 (reference)	1 (reference)
Mod + HEPA	25,293 (53.54)	163,062.08	2	1.23 (0.31–4.9)	0.2 (0.04–0.95)	0.19 (0.04–0.90)	0.28 (0.05–1.47)
CAC > 0	9227	52,068.76	24	46.09 (30.89–68.77)			
Inactive	3663 (39.70)	20,537.73	10	48.69 (26.2–90.49)	1 (reference)	1 (reference)	1 (reference)
Mod + HEPA	5564 (60.30)	31,531.03	14	44.4 (26.3–74.97)	0.81 (0.36–1.84)	0.77 (0.33–1.76)	0.56 (0.17–1.89)

Values are expressed as hazard ratios (95% confidence interval), number (frequency). CAC is expressed in Agatston unit. Abbreviations: CAC, coronary artery calcification; CI: confidence interval; HR: hazard ratio; inactive, physically inactive; Mod + HEPA, moderately active and health-enhancing physical activity; IPTW: inverse probability of treatment weighted. Model 1: adjusted for age, sex; Model 2: adjusted for sex, age, BMI, alcohol intake, smoking status, education level, systolic blood pressure, fasting glucose, serum LDL, history of heart disease, diabetes, dyslipidemia, and hypertension. IPTW: marginal structural model based on IPTW.

**Table 4:** Mortality rates based on physical activity levels and CAC: physically inactive without CAC as a reference.

	Number	Person-Years	Mortality	Morality Rate (per 10^5^ PY)	Model 1	Model 2	IPTW HR (95% CI)
Total		354,585.17	34	9.59 (6.85–13.42)			
Inactive and CAC = 0	21,949 (38.87)	139,454.32	8	5.74 (2.87–11.47)	1 (reference)	1 (reference)	1 (reference)
Inactive and CAC > 0	3663 (6.49)	20,537.73	10	48.69 (26.2–90.49)	3.84 (1.38–10.65)	3.60 (1.25–10.37)	2.81 (1.76–19.19)
Mod active + HEPA & CAC = 0	25,293 (44.79)	163,062.08	2	1.23 (0.31–4.9)	0.2 (0.04–0.94)	0.20 (0.04–0.94)	0.28 (0.05–1.45)
Mod active + HEPA & CAC > 0	5564 (9.85)	31,531.03	14	44.4 (26.3–74.97)	3.14 (1.17–8.46)	2.72 (0.97–7.66)	3.27 (1.14–9.38)

Values are expressed as hazard ratios (95% confidence interval), number (frequency). CAC is expressed in Agatston unit. Abbreviations: CAC, coronary artery calcium; inactive, physically inactive; Mod + HEPA, moderately active and health-enhancing physically active group; IPTW: inverse probability of treatment weighting. Model 1: adjusted for age and sex; Model 2: adjusted for sex, age, BMI, alcohol intake, smoking status, education level, systolic blood pressure, fasting glucose, serum LDL, history of heart disease, diabetes, dyslipidemia, and hypertension; IPTW: marginal structural model based on IPTW.

## Data Availability

The datasets generated during and/or analyzed during the current study are not publicly available but are available from the corresponding author on reasonable request.
